# Risks of suicide in migraine, non-migraine headache, back, and neck pain: a systematic review and meta-analysis

**DOI:** 10.3389/fneur.2023.1160204

**Published:** 2023-04-20

**Authors:** Alec Giakas, Kiersten Mangold, Anthony Androulakis, Noah Hyduke, Igor Galynker, Melinda Thiam, Guoshuai Cai, X. Michelle Androulakis

**Affiliations:** ^1^Department of Orthopedic Surgery, University of South Carolina School of Medicine, Columbia, SC, United States; ^2^Department of Exercise Science, University of South Carolina, Columbia, SC, United States; ^3^Department of Biological Sciences, University of South Carolina, Columbia, SC, United States; ^4^Department of Psychiatry, University of South Carolina School of Medicine, Columbia, SC, United States; ^5^Department of Psychiatry, Beth Israel Medical Center, New York, NY, United States; ^6^Department of Psychiatry, New Mexico VA Hospital System, Albuquerque, NM, United States; ^7^Department of Environmental Health Sciences, University of South Carolina, Columbia, SC, United States; ^8^Department of Neurology, Columbia VA Healthcare System, Columbia, SC, United States; ^9^Department of Neurology, University of South Carolina School of Medicine, Columbia, SC, United States

**Keywords:** suicide, chronic back pain, chronic neck pain, migraine, headache

## Abstract

**Objective:**

To conduct a systematic review and meta-analysis on suicidal ideation, attempts, and death in patients with head, neck, and back pain.

**Method:**

Search was performed using PubMed, Embase, and Web of Science from the date of the first available article through September 31, 2021. A random effects model was used to estimate the pooled odds ratios (ORs) and 95% confidence intervals (95% CI) for the association between suicidal ideation and/or attempt and head, back/neck pain conditions. Articles describing non-migraine headache disorders and death by suicide were also reviewed but not included in the meta-analysis due to an insufficient number of studies.

**Results:**

A total of 20 studies met criteria for systemic review. A total of 186,123 migraine patients and 135,790 of neck/back pain patients from 11 studies were included in the meta-analysis. The meta-analysis showed that the estimated risk of combined suicidal ideation and attempt in migraine [OR 2.49; 95% CI: 2.15–2.89] is greater than that in back/neck pain pain [OR 2.00; 95% CI: 1.63–2.45] compared to non-pain control groups. Risk of suicide ideation/planning is 2 folds higher [OR: 2.03; 95% CI: 1.92–2.16] and risk of suicide attempt is more than 3 folds higher [OR: 3.47; 95% CI: 2.68–4.49] in migraine as compared to healthy controls.

**Conclusion:**

There is an elevated risk of suicidal ideation and attempt in both migraine and neck/back pain patients in comparison to healthy controls, and this risk is particularly higher among migraine patients. This study underscores the critical need for suicide prevention in migraine patients.

## 1. Introduction

Chronic pain is a leading cause of disability worldwide and affects upwards of 20.4% of adults in the United States (1). Headache disorders, especially migraine, are among the most common types of chronic pain conditions encountered by neurologists and psychiatrists. Migraine has a global negative impact on overall quality of life, cognitive, emotional health, and contributes to isolation, frustration, guilt, fear, avoidance behavior, and stigma (2). Individuals with migraine thus often learn to internalize symptoms with strict concealment, as the manifestations of migraine, as well as its degree of severity and disability, are invisible: pain, cognitive impairment, nausea, vertigo, hypersensitivity to the environment, aura, etc. (3). Despite the prevalence and impact of migraines, 70% of migraine patients do not seek medical advice (4, 5).

Migraine has significant association with multiple mental health disorders. For example, bidirectional association exists between migraine and psychiatric disorders such as anxiety disorder, bipolar disorder, and depression (6–8). This is especially true for the military and Veteran population. In 2020, the suicide rate for Veterans was 57.3% greater than for non-Veteran U.S. adults when adjusted for demographic differences (9). Traumatic brain injury (TBI), PTSD, and depression are significant risk factors for chronic headache in Veterans (10). Veterans with chronic headache, especially those with comorbid TBI, PTSD, and depression, are at increased risk for suicidal behavior (10, 11). As such, there is a need to better understand the relationship between suicidality and head, neck, and back pain, and establishing interdisciplinary collaboration when caring for Veterans with migraine, which often go undiagnosed or not coded (12).

One cannot “split the brain”; therefore, improved understanding of increased suicide risk among headache disorders affords a unique opportunity for clinicians of various disciplines to proactively engage in dialogue to collaborate and advocate for better treatment. Currently, most of headache management is done by specialty care or primary care separately. We hope to bring awareness to the increased risk of suicide among headache disorders, which serves as an invitation for neurologists, psychiatrists, psychologists, and others involved in patient care to join the interdisciplinary care team.

Previous research has indeed associated migraines with an increased suicide risk (13, 14), but previous review and meta-analysis are lacking regarding the potential risk for suicide in both migraine and other pain disorders. There is also a lack of comprehensive review between different types of headache disorders and a spectrum of suicidal behaviors. As such, authors conducted a systematic review and meta-analysis to examine the risk of different types of suicidal behavior (i.e., ideation, attempts, and death) in patients with migraine, non-migraine headache conditions, and back/neck pain compared to healthy controls.

## 2. Methods

### 2.1. Literature search

Authors conducted the literature search using three databases (PubMed, EMBASE, and Web of Science) from the date of the first available article through September 31, 2021. Studies related to headache, back pain, or neck pain and suicide were identified using keywords “migraine AND suicide,” “cluster headache AND suicide,” “trigeminal autonomic cephalgia AND suicide,” “post-traumatic headache AND suicide,” “tension headache AND suicide,” “trigeminal neuralgia AND suicide,” “hemicrania continua AND suicide,” “chronic back pain AND suicide,” “back pain AND suicide,” “lumbar pain AND suicide,” “cervicalgia AND suicide,” “chronic neck pain AND suicide,” and “neck pain AND suicide.” No other filters were used. The principles of the Preferred Reporting Items for Systematic Reviews and Meta-Analyses was used to further screen and filter studies. One reviewer screened studies to determine whether they meet the eligibility criteria as outlined below, and one independent reviewer confirmed that only papers that fully met these criteria were included in this study. The full text of all included studies was then examined by a third reviewer to ensure that data had been recorded accurately.

### 2.2. Eligibility criteria

To be included in the analysis, studies were required to meet all of the following criteria: (A) participants were adults 18 years of age or older; (B) the study defined the type of suicidal behavior being examined; (C) the study used healthy controls or controls without chronic pain conditions; (D) the study assessed the association of an individual headache disorder (migraine, cluster headache, tension headache, trigeminal autonomic cephalgia, and trigeminal neuralgia) or chronic back or neck pain with suicidal behavior or death by suicide as compared to healthy controls; (E) the study was published in the English language.

Studies were excluded if they met the following criteria: (A) inclusion of participants under 18 years of age; (B) published in the form of conference abstracts/posters, editorials, guidelines, or reviews; (C) insufficient data, such as raw data, mean or *p*-value; (D) lack of a healthy control group; (E) patients with chronic pain included in the control group.

### 2.3. Data collection and analysis

Data were extracted by the same researcher from each article and included the following items: year, population of interest, ICD codes and/or method of defining chronic pain, statistical method, sample size of the chronic pain and healthy control groups, odds ratio of risk of suicide in pain group compared to healthy control group, and confidence intervals. Unadjusted ORs were used due to inter-study variance in controlled factors. Authors used Comprehensive Meta-Analysis (CMA) V3 software (Biostat, NJ, USA) to generate summary statistics and pooled adjusted ORs using a random effects model, as stratified by the suicidal behavior (suicide ideation, suicide attempt) and pain types (migraine, chronic neck/back pain).

## 3. Results

**Figure 1:**
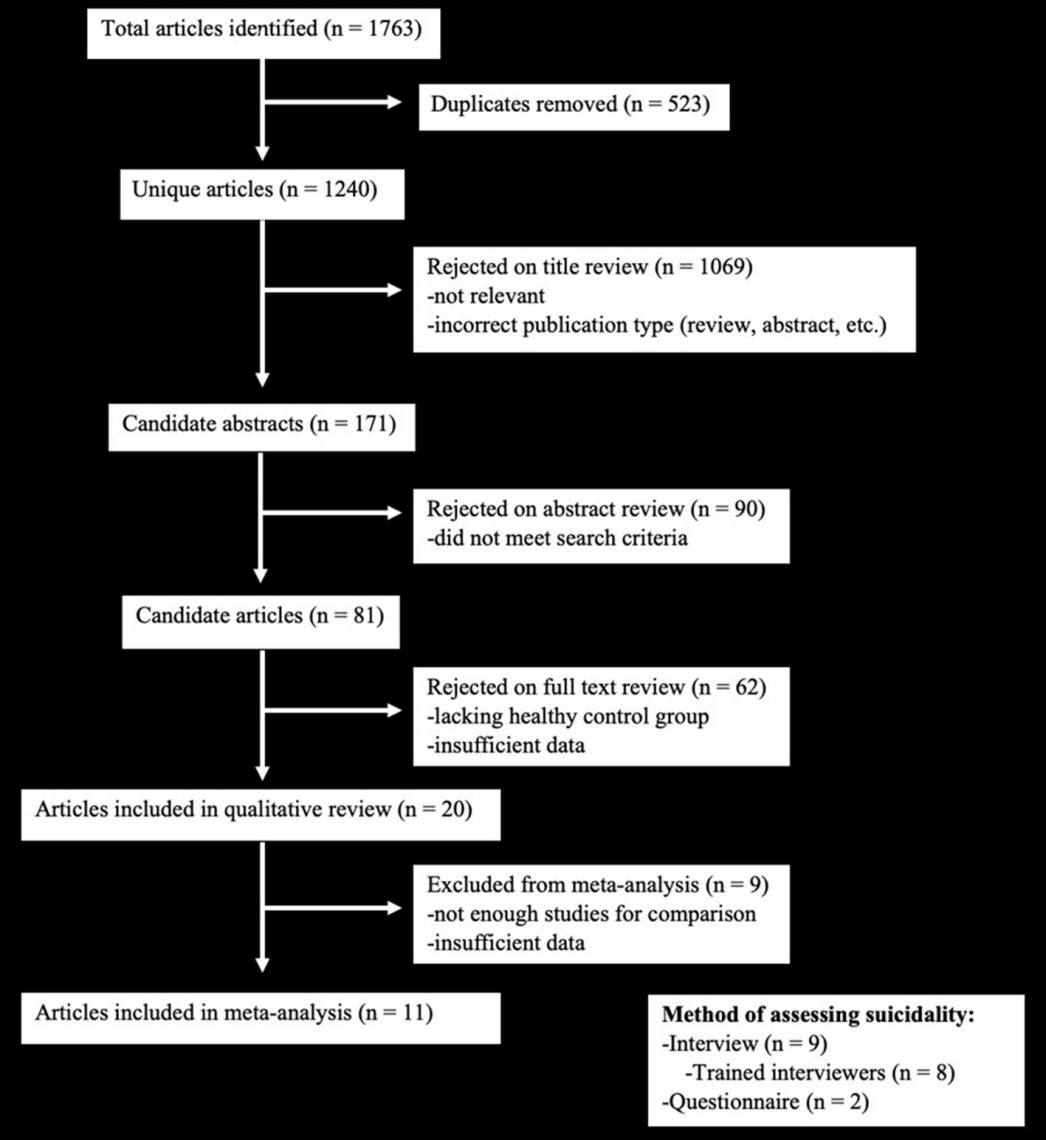
PRISMA inclusion diagram for meta-analysis, which demonstrates the process through which the studies used in the manuscript were selected from the total number of studies identified.

Initial search yielded 1,763 results, which were then screened for inclusion in the review and meta-analysis (Figure 1). Twenty studies met the criteria to be included in the systematic review. Two of these studies examined cluster headache disorder (15, 16) and two examined tension-type headache (17, 18) so meta-analysis was not conducted for these specific headache disorders due to limited study numbers. Similarly, only two studies examined death by suicide as an outcome, they were not included in the meta-analysis (19, 20). The meta-analysis was therefore conducted for migraine and back/neck pain with suicidal ideation/planning or suicide attempts as outcome measures. Three additional studies were lacking specific statistics and thus were not eligible for the meta-analysis (21–23). Therefore, a total of 11 studies were included in the meta-analysis.

### 3.1. Meta-analysis results

**Figure 2:**
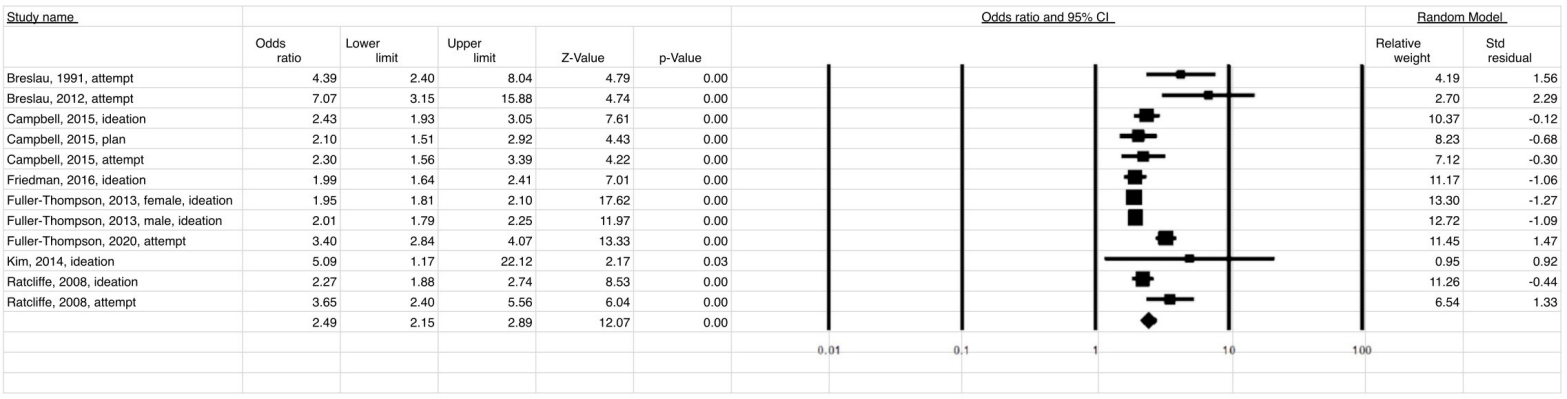
Relationship between suicide ideation and attempt (OR = 2.49) in patients with migraine when compared to healthy controls.

**Figure 3:**
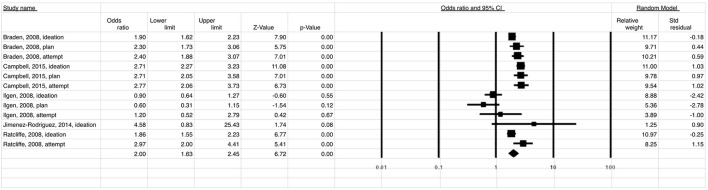
Relationship between suicide ideation and attempt (OR = 2.00) in patients with back/neck pain when compared to healthy controls.

**Figure 4:**
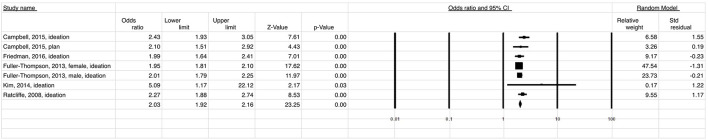
Relationship between the risk of suicide ideation/planning (OR = 2.03) in patients with migraine when compared to healthy controls.

**Figure 5:**
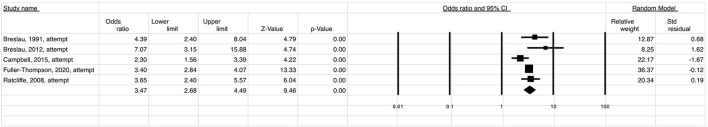
Relationship between suicide attempt (OR = 3.47) in patients with migraine when compared to healthy controls.

**Figure 6:**
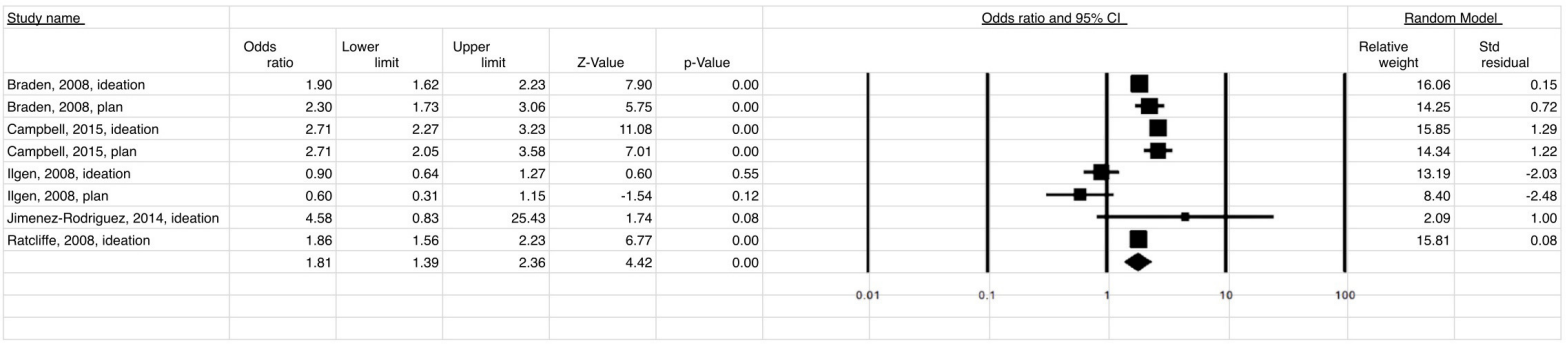
Relationship between risk of suicide ideation/planning (OR=1.81) in patients with back/neck pain when compared to healthy controls.

**Figure 7:**
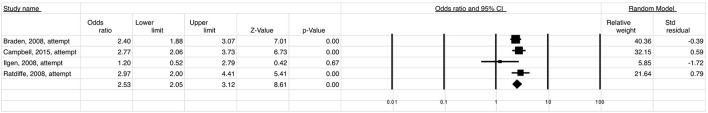
Relationship between risk of suicide attempt (OR = 2.53) in patients with chronic back/neck pain when compared to healthy controls.

A total of 186,123 migraine patients and 135,790 of back/neck pain patients were included in the meta-analysis. The meta-analysis showed that the estimated risk of suicidal ideation and/or attempt both in patients with migraine (OR 2.49; 95% CI: 2.15–2.89) (Figure 2) and in those with back/neck pain (OR 2.00; 95% CI: 1.63–2.45) (Figure 3) was significantly elevated when compared to healthy controls. Migraine was associated with a 2-fold higher risk of suicidal ideation/planning (OR: 2.03; 95% CI: 1.92–2.16) (Figure 4) and over three times higher risk of suicide attempt (OR: 3.47; 95% CI: 2.68–4.49) (Figure 5) when compared to controls. The odds of suicidal ideation/planning in back/neck pain were just under two times that of healthy controls (OR: 1.81; 95% CI: 1.39–2.36) (Figure 6). Chronic back/neck pain was associated with a 2.53 (95% CI: 2.05–3.12) times increased odds of suicide attempts (Figure 7), which was lower than that associated with migraine (*z* test, *p* = 0.04).

### 3.2. Reviews of studies not included in meta-analysis

#### 3.2.1. Death by suicide

Two studies identified in the present review examined death by suicide as an outcome measure. One study examined the association between migraine and both self-harm and suicide mortality. The authors reported an increased odds (hazard ratio = 2.18) of self-harm for those diagnosed with migraine but did not find an association between migraine and death by suicide (19). However, another study reported an increased risk of death by suicide associated with migraine (hazard ratio = 1.68), which persisted when controlling for psychiatric comorbidities (hazard ratio = 1.34) (17).

#### 3.2.2. Non-migraine headache disorders

Three studies identified in our search examined suicidality in relation to headache disorders distinct from migraine, specifically cluster headache and tension headache. Cluster headache was reported to increase the risk of suicidal ideation by 2.5 times [OR: 2.49, 95% CI: 1.91–3.25] (16) and around 2-fold [OR: 2.04, 95% CI: 1.08–3.85] (15) when compared to controls in two separate studies, even after accounting for depression and demoralization. A similar pattern was reported for tension headaches, which were associated with 2.39 times higher odds of suicidal ideation or attempt, defined as suicidal ideation and/or attempts (18). One study also reported an increased risk of death by suicide associated with headache or tension headache (hazard ratio = 1.38) (17). When distinguishing between chronic and episodic tension headache, only chronic tension headache was associated with increased suicidal ideation or attempt (18). In further comparison of headache conditions, another study reported an increased odds of suicide attempt for both migraine [OR: 7.21, 95% CI: 3.21–16.2] and non-migraine headache [OR: 8.38, 95% CI: 3.35–21.0] when compared to controls with no history of severe headache; however, there were no differences between migraine and non-migraine type headaches for odds of suicide attempts (24).

## 4. Discussion

This study sought to examine the risk of suicidality among patients suffering from migraine, non-migraine headache disorders, and back/neck pain. This meta-analysis demonstrates that suicidal ideation and attempts are both significantly increased among patients with migraine or back/neck pain compared to healthy controls, whereas systematic review demonstrates an increased risk of suicidality in patients with non-migraine headache disorders as well. Of note, the risk of suicide attempt in patients with migraine is statistically higher than in those with back/neck pain. The odds associated with suicide attempts in migraine were over 3-fold—the highest for any of the pain conditions and suicidal behaviors investigated in this study.

Previous literature reviews have described an increase in suicidal ideation and attempts associated with non-specific chronic pain (25–28). Our study provides additional evidence for increased risk of suicidality in patients with migraine and non-migraine headache disorders. Most importantly, our results shed light on a potentially higher risk of suicide attempt in patients with migraine than in those with back/neck pain.

It is worth noting that studies examining the association between mental health disorder diagnosis as it relates to chronic pain and suicidality reveal inconsistent results. Some studies suggested that the relationship between migraine or back/neck pain and suicidality persists when accounting for psychiatric comorbidities (24, 29–31) while others didn't find significant associations (21, 32, 33). Further discrepancies are present based on the type of suicidal behavior examined. For example, one study reported that, when controlling for mental health disorders, migraine remained associated with suicidal ideation but not planning or attempts (30), whereas another study noted a consistent association with suicidal ideation and planning but not attempts, which may be due to under diagnosis of mental health disorders (34). Regardless of the intensity of pain of migraine attacks, the frequency and duration have been found to have a strong association with the burden of psychiatric comorbidity [OR: 7.21, 95% CI: 3.21–16.2] (6).

It is imperative to note while migraine is strongly associated with elevated suicide risk, physicians should also be familiar with increased suicide risk in patients with cluster and chronic tension headaches. Very few studies examined non-migraine type headache disorders, and thus were not included in the meta-analysis. However, upon systematic review, the odds of self-inflicted injury, suicidal ideation and attempt, or death by suicide for cluster headache and chronic tension headache were similar to that of migraine (15–18). Cluster headaches have been linked with increased suicidal ideation, planning and attempts during attacks, which was found to predict increased suicidality in the interictal phase of cluster headache. In addition, longer disease burden, even with episodic cluster headaches, was associated with a similar increase in suicidality, prompting the need for preventative treatment (35). These results further parallel those of another study showing the risk of suicide attempts in migraine patients was equivalent to that in non-migraine type headaches (24).

Suicide Crisis Syndrome, or SCS, is a recently described acute suicidal mental state which may link migraines and suicidal behavior (36). Several SCS criteria have symptoms overlapping with migraine (36, 37). Of note, CDC report demonstrated that only a fraction of deaths by suicide expressed ideation before their death; >75% explicitly denied suicidal ideation prior to their death (38). One of the reasons for non-disclosure of suicidal ideation is that explicit suicidal intent may last less than 10 min preceding a suicide attempt, a very short period which is likely to occur outside the clinical setting. Another reason is that suicidal individuals would not admit to their suicidal intent out of fear of being hospitalized and losing their autonomy; this is potentially more likely to occur among military personnel and Veterans. SCS, on the other hand, is diagnosed indirectly, without asking about suicidal ideation or intent, and has consistently outperformed suicidal ideation for prediction of future suicidal behavior (13–16, 39). It is worth noting that all studies reviewed used a simple yes/no question to elicit suicidal behavior (Table 1). This represents a significant void in clinical research studying associations between migraine and suicidal behavior. Using SCS checklist may be an exceedingly useful tool for identifying individuals with migraine headaches who are at imminent risk for suicide.

**Table 1:** Clinical features assessed in meta-analysis articles.

**References**	**Method**	**Diagnostic criteria of pain condition**	**Frequency/chronicity of pain**	**Trained interviewer**	**Suicidality assessment details**
Braden and Sullivan (33)	In-person interview for all questions other than suicidality. Suicidality questions were asked in a self-administered booklet rather than interview	Self-reported endorsement of: arthritis/rheumatism, chronic back or neck problems, frequent or severe headaches, or “other” chronic pain	Frequency: not specified Chronicity: lifetime and in the past 12 months	Yes	Stand-alone questions regarding if they had “ever seriously thought about committing suicide, made a plan for committing suicide, or attempted suicide”
Breslau et al. (40)	In-person interview	Criteria adapted from the 1988 Headache Classification Committee of the International Headache Society diagnostic criteria for migraine	Frequency: at least 5 occurrences for classification of migraine Chronicity: lifetime prevalence	Unspecified	Single question on suicide attempts from National Institute of Mental Health's Diagnostic Interview Schedule
Breslau et al. (24)	In-person interview	Migraine: features from the ICHD-2 criteria Non-migraine severe headache: duration of >4 h, no history of migraine-like features, and minimum Headache Impact Questionnaire score of 38.05	At least 1 headache in the past year	Yes	Single question on suicide attempts from World Health Organization Composite International Diagnostic Interview
Campbell et al. (30)	In-person interview	WMH-CIDI with questions pertaining to arthritis, migraines, and neck/back pain	Frequency: not specified Chronicity: pain condition persisted for 6 months	Yes	Multiple questions on suicidal behavior (ideation, plans, attempts) from WMH-CIDI
Friedman et al. (31)	In-person interview	Migraine: meeting ICHD-III beta criteria administered in a Spanish-language questionnaire Probable migraine: Meet all but one of the migraine criteria	Frequency: not specified Chronicity: not specified	Yes	Suicidal ideation item from the Patient Health Questionnaire-−9
Fuller-Thompson et al. (41)	In-person interview	Self-report of having previously been diagnosed with migraine by a health professional	Frequency: not specified Chronicity: migraine had persisted or was expected to persist for 6 months or more	Yes	One stand-alone question on suicidal ideation: “have you ever seriously considered committing suicide or taking your own life?”
Fuller-Thompson and Hodgins, (42)	In-person interview	Migraine: self-report of having previously been diagnosed with migraine by a health professional Chronic pain: self-report of usual pain or discomfort that is of moderate or severe intensity	Frequency: not specified Chronicity: migraine had persisted or was expected to persist for 6 months or more	Yes	Single question on suicide attempts asking “if they had ever “attempted suicide or tried to take (their) own life”
Ilgen et al. (17)	In-person interview	A series of yes/no questions pertaining to: arthritis or rheumatism, chronic back or neck problems, frequent or severe headaches, and any other chronic pain	Frequency: not specified Chronicity: 12-month prevalence	Yes	Stand-alone questions regarding suicidal ideation, plans, and attempts (both lifetime and in the last 12 months)
Jimenez-Rodríguez et al. (43)	Questionnaire	Constant or intermittent non-specific low back pain	Frequency: not specified Chronicity: at least the past 3 months	N/A	Suicidal ideation assessed with question 9 of the Beck Depression Inventory and risk of suicide with the Plutchik Suicide Risk Scale
Kim and Park (32)	In-person interview	Revised version of the ICHD-II	Chronic migraine was defined as frequency of ≥15 headache days per month in the prior 3 months, with at least 8 days per month meeting the criteria for migraine without aura. Those not meeting this definition were included as non-chronic migraine. Migraine patients were included as a single group in the analysis, regardless of chronicity.	Yes, trained neurologist	Beck scale for suicidal ideation
Ratcliffe et al. (29)	In-person interview	Self-report of chronic pain conditions (i.e., arthritis or rheumatism, back problems, migraine headaches, fibromyalgia) previously diagnosed by a health professional	Frequency: not specified Chronicity: condition had persisted or was expected to persist for 6 months or more	Yes, professional interviewers with “additional training to increase sensitivity on mental health issues”	Stand-alone questions regarding suicidal ideation and attempts (both lifetime and in the last 12 months)

Apart from Ratcliffe et al. (29), the type of training that interviewers received was unspecified (i.e., whether training was general to interview administration or specific to mental health). Although Fuller-Thomson et al. (41) and Fuller-Thompson and Hodgins (42) do not specify training type, data were obtained from the same national survey as Ratcliffe et al. (29) (i.e., the Canadian Community Health Survey), albeit from different years. ICHD, International Classification of Headache Disorders; WMH-CIDI, World Mental Health Survey Initiative version of the Composite International Diagnostic Interview.

Despite the novelty of this study, there are some limitations. As discussed above, there were very few studies that included patients with headache disorders other than migraine, thus we were only able to include patients with migraine and back/neck pain in the meta-analysis. This lack of existing literature limits the conclusions we are able to draw pertaining to non-migraine type headache disorders and highlights an important direction for future research to allow for broader and more detailed analysis of suicidality in headache disorders. Additionally, our meta-analysis pooled results were uncontrolled for different variables, such as psychiatric comorbidities. We chose to analyze uncontrolled results because of the variability between studies in their adjusted analyses. For example, some studies controlled for diagnosed psychiatric disorders (24, 33, 40), while others controlled for self-reported depression or anxiety scores (32) and adverse childhood experiences (42).

A national cohort study in the Veterans Health Administration for fiscal year 2008–2019 found that Veterans with headache were being seen at the emergency department at the same rate as at neurology clinics and at a larger rate than headache specialists (12). In patients who died by suicide, it has been elucidated that one-third had contact with mental health services within a year and one-fifth had contact within a month of their suicide. Additionally, about three-quarters of patients were seen by their primary care physician within a year, and about one-half of patients were seen within the month, of their suicide (39). This further demonstrates the need for increasing awareness of suicide risks among Veterans with different types of pain conditions and for collaboration between specialties within VHA.

## Data availability statement

The raw data supporting the conclusions of this article will be made available by the authors, without undue reservation.

## Author contributions

XMA contributed to the design, interpretation of results, and drafting of the study. All authors contributed to the interpretation of results, drafting, and the revision of the final manuscript.
